# Case Report: A rare pediatric case of paraneoplastic pemphigus associated with Castleman disease misdiagnosed as Behçets disease

**DOI:** 10.3389/fped.2024.1469495

**Published:** 2024-11-22

**Authors:** Ranran Zhang, Jia Liu, Nana Nie, Dahai Wang, Jie Wu, Huanyu Zhang, Ruiyun Zhang, Shan Gao, Cui Bai, Yi Lin, Qiuye Zhang, Hong Chang

**Affiliations:** ^1^ Department of Pediatric Nephrology, Rheumatology and Immunity, The Affiliated Hospital of Qingdao University, Qingdao, China; ^2^ Department of Pathology, The Affiliated Hospital of Qingdao University, Qingdao, China; ^3^ Department of Pediatric Surgery, The Affiliated Hospital of Qingdao University, Qingdao, China; ^4^ Department of Pediatrics, Qingdao Municipal Hospital, Qingdao, China

**Keywords:** paraneoplastic pemphigus, Castleman disease, oral ulcers, early diagnosis (MeSH), pediatric

## Abstract

Castleman disease (CD) is a rare lymphoproliferative disease known as angiofollicular lymph node hyperplasia, firstly reported in 1954. It mainly occurs in adults, presenting with a wide range of clinical manifestations, including paraneoplastic pemphigus (PNP). PNP is a rare and often life-threatening autoimmune disorder characterized by painful blisters and erosions on the skin and mucous membranes. In children, PNP is often linked to Castleman disease, as evidenced in case reports. So far, less than 30 pediatric cases have been reported, with the pathogenesis remaining unclear and treatment approaches varied. Here, we present a pediatric case initially suspected as Behçet's disease due to persistent oral ulcers and conjunctivitis, and undergone a sudden aggravation of clinical features following an allergic reaction. New involvement of skin rashes and imaging findings prompted the final diagnosis as PNP linked to Unicentral Castleman disease (UCD).Through detailing the progression of clinical features and diagnostic work, we aim to arise the awareness of physicians and put emphasize on early recognition and multidisciplinary management, which can improve patient outcomes.

## 1 Introduction

PNP is a rare autoimmune blistering disease characterized by intractable mucositis, diverse skin lesions, acantholysis, and lichenoid or interface dermatitis, originally proposed by Dr Anhalt in 1990 (1, 2). It is often linked to underlying neoplasms, notably hematologic and lymphoproliferative disorders like Castleman disease. Castleman disease can be divided into two types: unicentric Castleman disease (UCD) and multicentric Castleman disease (MCD). It can also be classified histologically into hyaline vascular and plasmacytic variants (3). Unlike in adults, paraneoplastic pemphigus in children was mostly caused by UCD. Globally, there have been approximately 30 reported pediatric cases of PNP combined with CD, most of which were individual case reports (4, 5). The causes of the disease remain unclear, but it is suspected that self-antibodies secreted by Castleman disease tumors lead to the skin lesions. Here, we aim to offer new insights by providing a detailed description of a patient with paraneoplastic pemphigus and Castleman's disease. Additionally, we will draw on a review of relevant literature to support our findings and recommendations.

## 2 Case presentation

### 2.1 Present history

**Figure 1:**
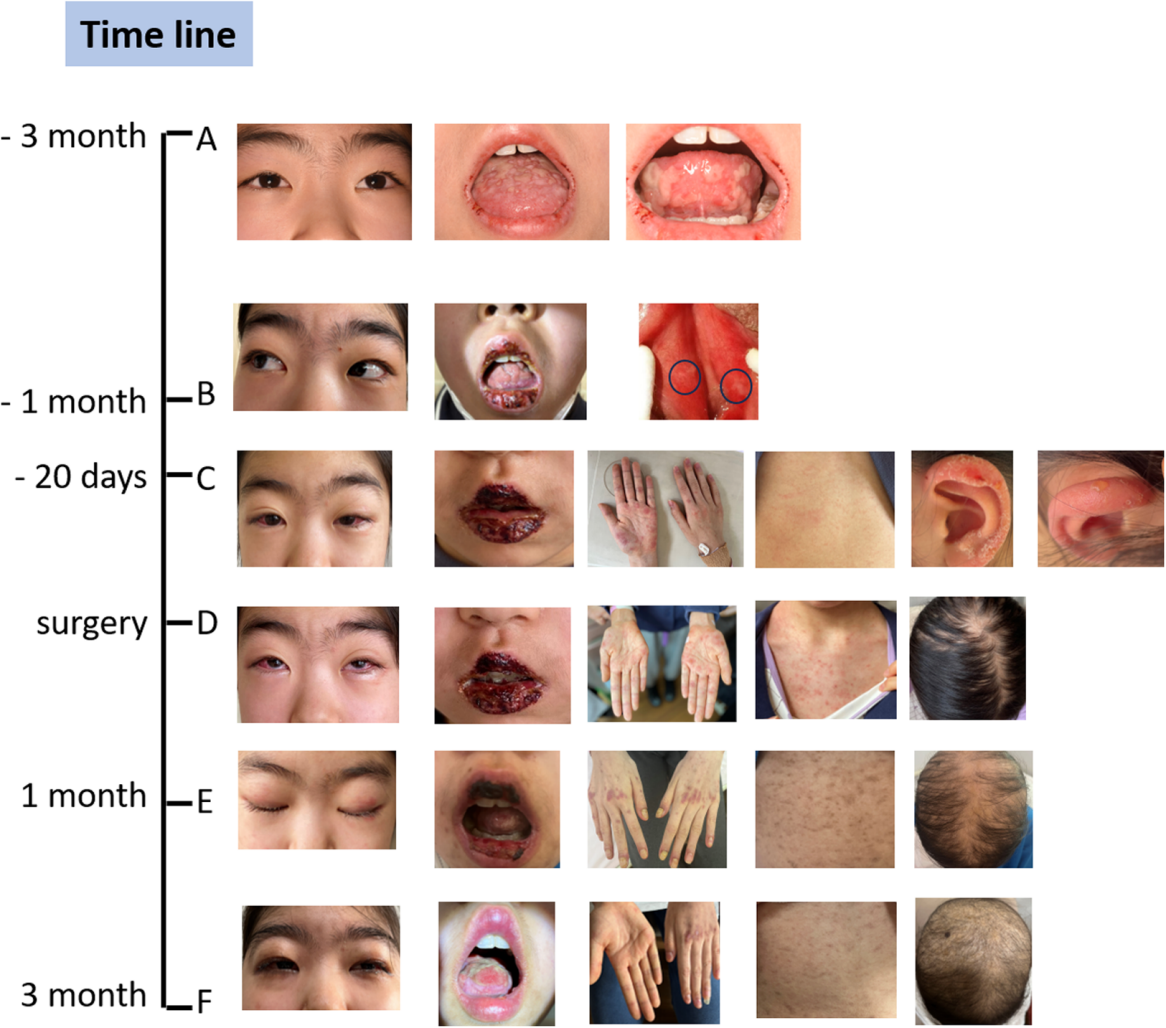
The spectrum of changes in clinical symptoms over time. **(A)** The symptoms were very mild at the onset of the oral ulcers. **(B)** The symptoms worsened upon admission to our hospital, and we observed slight perineal ulcers. **(C)** The symptoms rapidly worsened after the use of antiviral drugs, which caused an allergic reaction, resulting in rashes all over the body, several blisters on the ears, and hair loss. **(D)** The clinical conditions at the time of deciding to proceed with surgery. **(E)** One month after the surgery, the oral ulcers and skin rashes had alleviated, but the conjunctivitis persisted, alopecia areata worsened, and nail dystrophy and onycholysis were observed. **(F)** Three months later, conditions including conjunctivitis, skin rashes, and oral ulcers had significantly improved, and new nails and hair were growing very slowly. The black circles in B panel pointed to perineal ulcers. She was healthy before the onset.

A 10-year-old girl was admitted to our hospital with recurrent oral ulcers lasting more than three months. Her main symptoms were painful oral ulcers (Figure 1A). These ulcers gradually expanded and merged, causing an uneven tongue surface, limited mouth opening, and restricted tongue extension. Additionally, the child presented with redness and itching in both eyes (Figure 1A), but no fever, cough, or other accompanying symptoms were noted. She has received treatment outside the hospital with topical medications, antiviral therapy, anti-allergy medications, and oral corticosteroids (15 mg daily) but failed to get satisfactory results as the ulcers continued to enlarge, affecting the patient's ability to eat. Subsequently, the patient received five injections of dexamethasone and betamethasone compound into the tongue muscle (administered every two days) at another hospital, which led to a slight improvement of the ulcers and relief of the pain. Given the challenges in treating the ulcers, their tendency to recur, and the negative results for antibodies related to blistering skin disease, “Behçet's disease” was the first suspicion, leading to a referral to our hospital.

### 2.2 Clinical findings and extensive diagnostic work-up

Upon admission, physical examination revealed swollen lips with fissures and blood crusts, a swollen and ulcerated tongue with an uneven surface, and multiple ulcerations in the oral mucosa with marked tenderness (limited mouth opening, approximately two finger-widths) (Figure 1B). Mild ulceration was also observed on the inner side of the labia minora, with multiple tender ulcerated areas palpable on the labia minora (Figure 1B).

Laboratory tests, including routine blood, urine, and stool tests, biochemical indicators, and inflammation markers, were all normal. Immunological tests revealed an antinuclear antibody titer 1:1,000, while other autoantibodies tested negative. The bullous skin disease antibodies, including desmoglein-3 antibody, anti-desmoglein-1 antibody, anti-BP180 antibody, and anti-BP230 antibody test results are all within the normal range. Imaging studies, including cardiac ultrasound, abdominal ultrasound, vascular ultrasound, and cranial MRI, showed no abnormalities.

Based on the 2013 International Behçet's Disease Classification Diagnostic Criteria, the patient received a score of 4 points for genital ulcers and 2 points for oral ulcers, suggesting a strong possibility of Behçet's disease. Following this diagnosis, the patient was initially treated with oral colchicine for ten days, along with local care for the eyes, mouth, and perineum. Antibiotics was also administered to prevent infections. The patient was managed with a multidisciplinary approach involving dermatologists, dentists, and ophthalmologists. The oral ulcers got more severe on the fourth day of admission, so methylprednisolone sodium succinate was added for anti-inflammatory treatment (25 mg, twice a day for eight days). One week after admission, TORCH antibody testing revealed positive IgM antibodies for herpes simplex virus type I/II, indicating a strong likelihood of herpes simplex virus infection. High-throughput genetic testing for localized pathogens identified a relatively high abundance of human beta herpesvirus 7. Intravenous immunoglobulin (IVIG) was administered at 10 g, and acyclovir was added for antiviral treatment. The patient also developed a cough, fever, and rhinorrhea, prompting the usage of antibiotics. Four days later, the patient developed generalized red, papular rashes with significant itching during intravenous acyclovir infusion (Figure 1C). This was considered an allergic reaction, leading to the discontinuation of acyclovir and its replacement with adenosine arabinose monophosphate. Ten days later, blisters were observed on the ears (Figure 1C).

**Figure 2:**
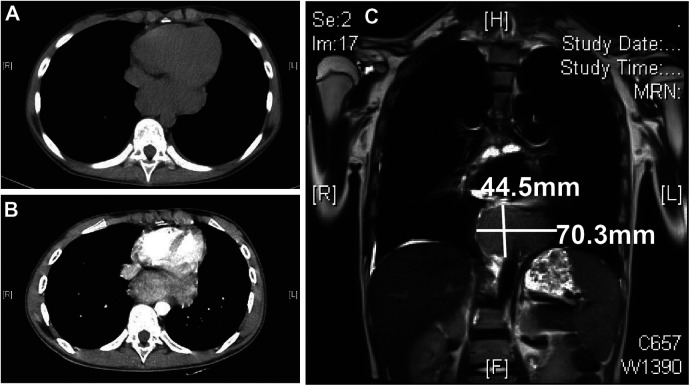
Chest CT and mediastinal MR results. Chest CT plain scan **(A)** and enhanced scan **(B)** both show a posterior mediastinal mass. Mediastinal MR examination **(C)** shows the mass size to be approximately 4.5 × 7.0 cm. CT, computer tomography; MR, magnetic resonance imaging.

**Figure 3:**
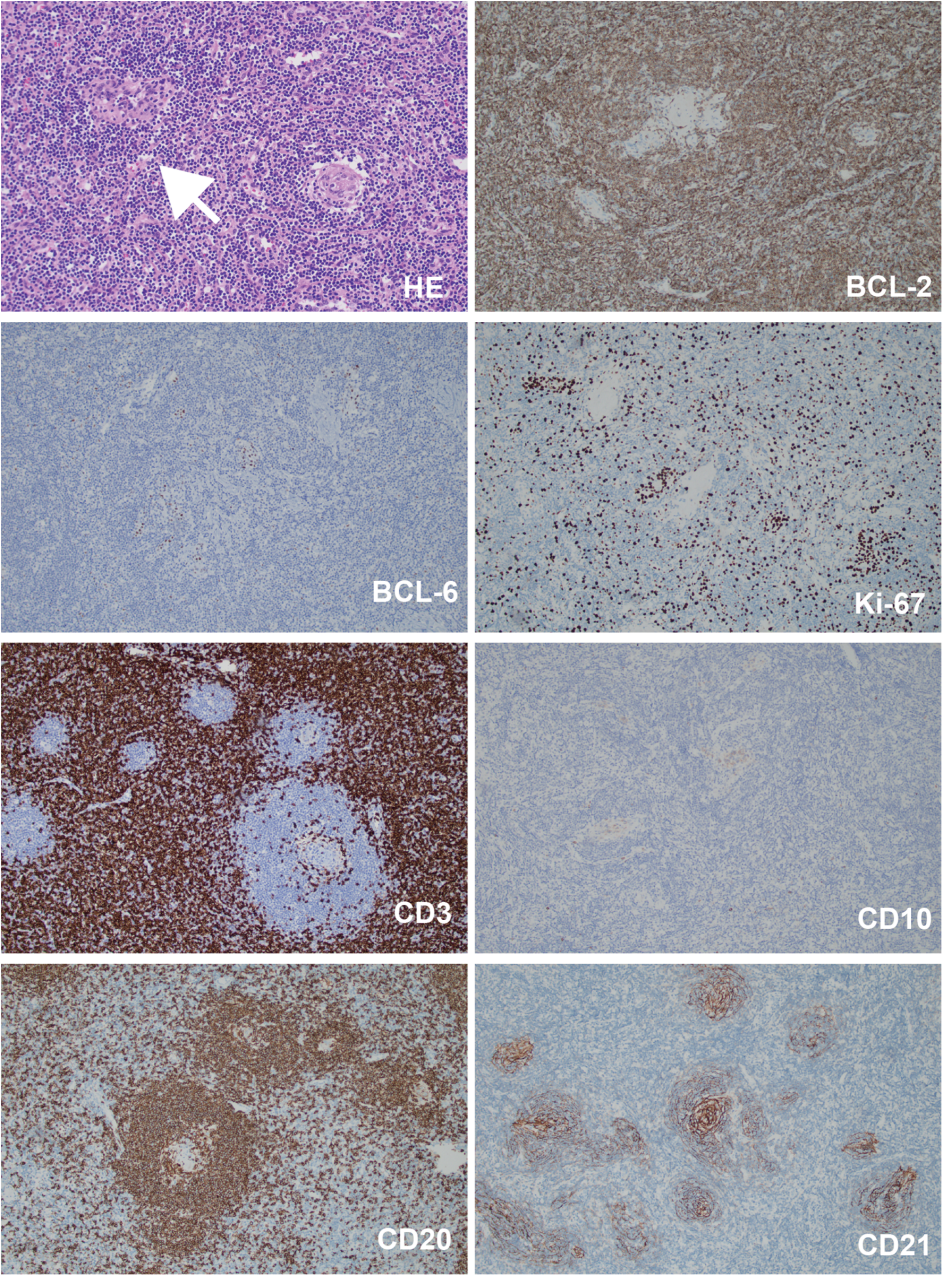
The histopathological staining results of the mediastinal mass. Bcl-2 (germinal center -), Bcl-6 (germinal center +), Ki-67 (+, 80% in the germinal center, 20% elsewhere), CD3 (T cells +), CD20 (B cells +), CD10 (germinal center +), CD21 (follicular dendritic meshwork +). White arrow pointed to the sclerotic blood vessel adjacent to the germinal center. Magnification: 200×.

The patient also developed a fever and worsening cough. An auxiliary examination tested positive for mycoplasma pneumoniae, promping the addition of azithromycin therapy for infection control. Consequently, a chest Computer Tomography (CT) scan revealed a mass adjacent to the lower esophagus in the mediastinum, along with a small amount of pleural effusion on both sides (Figure 2A). Further dynamic contrast-enhanced chest CT (Figure 2B) and mediastinal MRI (Figure 2C) were conducted, indicating a space-occupying lesion near the lower esophagus, suggesting a tumor-like lesion, potentially Castleman's disease. Taking into account the patient's history, clinical manifestations, and examination results, the girl was suspected to have Castleman's disease along with secondary pemphigus. Following a multidisciplinary team discussion, the patient received an endoscopic ultrasonography-guided fine needle aspiration that ruled out malignant tumors. Subsequently, she had video-assisted thoracoscopic surgery to remove the posterior mediastinal mass. The postoperative pathology revealed lymphoid hyperplasia, which is consistent with the hyaline-vascular type of Castleman's disease, based on the morphology, immunohistochemistry results, and molecular testing (Figure 3). However, the girl was in poor condition during the perioperative period (Figure 1D), and her parents refused to conduct skin or mucosal biopsies to verify the diagnosis of paraneoplastic pemphigus.

## 3 Follow-up care and outcome

Within one months after the surgery, the girl experienced worsening hair loss, transient worsening of the rash, and nail shedding (Figure 1E). To improve her clinical symptoms, the child received corticosteroids as immunosuppressive therapy and collaborative supportive treatments from oral physicians, ophthalmologists, and immunologists. Three months later, the girl got significant improvement of the oral ulcers, skin rashes, and new nails and hair grew (Figure 1F). Notably, within three months of postoperative follow-up, some immune indicators worsened before improving, despite the surgery (Table 1). These included changes in IL-6 levels, ANA titer, and the ratio of lymphocyte subsets, which explained temporary worsening of the clinical symptoms. Over time, the self-antibody titer decreased, and the condition of IL-6 level elevation and the CD4/CD8 inversion was corrected. However, despite the alleviation of cutaneous symptoms, the girl developed dyspnea after exercise three months post-operation. Chest CT scan showed unevenly decreased interstitial transparency in both lungs, and the pulmonary function test revealed severe restrictive ventilatory impairment, indicating the possibility of early obstructive bronchiolitis. Consequetly, the patient received two doses of rituximab (375 mg/m^2^ per week), along with preventive anti-infective measures and intravenous immunoglobulin (IVIG) therapy to support her immune system. However, the girl still got dyspnea after exercise, calling for more intensive supportive care and close follow-up.

**Table 1:** Several laboratory indicators before and after surgery.

	1 month before surgery	1 month after surgery	3 months after surgery	Reference range	Unit
Titer of ANA	1:1,000	1:3,200	1:1,000	Negative	-
IL-6	1.82	25.96	5.68	0–5.4	pg/ml
WBC count	5.01	7.83	6.49	4.3–11.3	×10^9^ cells/L
Neutrophil percentage	58.7	87.1	62.4	31.0–70.0	%
Lymphocyte percentage	29.7	10.9	29.0	23.0–59.0	%
CD4 T cell percentage	28.35	16.69	21.67	31.5–41.6	%
CD8 T cell percentage	35.71	37.52	37.01	18.1–29.6	%
B cell percentage	17.06	14.69	9.34	7.3–18.2	%
CD4/CD8 ratio	0.79	0.44	0.59	0.98–1.94	–
CD4 T cell count	422.415	141.865	761	621–1,258	cells/ul

ANA, antinuclear antibodies; IL, interleukin; WBC, white blood cell.

## 4 Methods and materials

Serum antibodies related to herpetic dermatosis were detected using an enzyme-linked immunosorbent assay (ELISA), and antibodies against desmogleins 1 and 3, as well as BP180 and BP230, were purchased from EUROIMMUN, China. A next-generation sequencing test was conducted on microbial cell-free DNA extracted from secretions of the eyes, mouth, and vagina by KnoinDX, China.

## 5 Discussion

PNP induced by Castleman disease is extremely rare and primarily documented through case reports. Persistent and painful mucosal involvement, expecially oral erosions, significantly affects the patient's quality of life and poses a therapeutic challenge. This case, initially suspected as Behçet's disease and finally diagnosed as PNP with CD, underscores the complexities involved in diagnosing and managing conditions that present with overlapping symptoms. The initial presentation of recurrent oral ulcers led to a suspicion of Behçet's disease, which is a common consideration in pediatric patients with similar symptoms to standard treatments over several months. However, the presence of generalized rashes and blisters following an allergic reaction and the discovery of a mediastinal mass led to the diagnosis of Castleman's disease with PNP. This highlights the importance of the broad differential diagnosis when faced with atypical presentations, particularly in children. What's more, we tried to dig out more pathophysiological findings through illustrating the dynamic changes of the clinical features.

### 5.1 Clinical presentation and diagnostic challenges

Paraneoplastic pemphigus is an autoimmune skin disorder associated with malignancies, particularly hematologic neoplasms such as non-Hodgkin lymphoma, chronic lymphocytic leukemia, and other conditions (6). PNP mainly manifested as the presence of mucocutaneous lesions, such as blisters, erosions, and ulcerations in the mouth, skin, and other mucosal surfaces. PNP induced by Castleman disease is a scarce condition, often presenting with only oral ulceration for several months before finding the potential tumors. Current literature pointed out that almost all pediatric patients with PNP experienced a period of misdiagnosis of more than 3 months until the eventual discovery of the tumor (4).

Persistent oral ulcers without skin manifestations may be linked to canker sores, Behçet's disease, nutritional deficiencies, infections (HIV, Epstein-Barr virus, cytomegalovirus, tuberculosis, toxoplasmosis, etc.), certain immune deficiency disordrs, and neoplastic disorders (7). Especially, the presence of two or more sites of mucosal ulcers can often lead to a misdiagnosis of Behçet's disease. Early biopsy and immunological testing are crucial for an accurate diagnosis. Additionally, effective management of PNP involves treating the underlying tumor, as demonstrated in the patient with Castleman disease. However, the differential diagnositic process was highly complicated due to polymorphic skin lesions (8).

Some patients also present systemic symptoms, such as night sweats, fever, weight loss, and fatigue, reflecting these conditions' underlying inflammatory and immune dysregulation (9). What is more, organ involvement, particularly renal dysfunction, hepatosplenomegaly, and lymphadenopathy, may occur in patients with Castleman disease and PNP and require tailored treatment strategies (9). The pulmonary involvement was observed in most patients (81.2%), while bronchiolitis obliterans (BO) was irreversible and possibly the main cause of death (5).

Diagnosing PNP involves clinical suspicion corroborated by histopathological and immunopathological findings. In this case, early suspicion and recognition were much more critical. However, her parents refused to perform skin biopsies to verify the diagnosis of PNP, since she was in poor condition when finding out the mediastinal mass. Nevertheless, based on the similar mucocutaneous features of PNP reported in literature and the subsequent response to operation and immunotherapy, it is clinically clear that the childs symptoms are consistent with CD-induced PNP. This case highlights the importance of being vigilant fot the possibility of PNP in children with severe oral ulcers and thoroughly investigating for any signs of tumors.

### 5.2 Pathophysiological insights

The coexistence of PNP with Castleman disease highlights the intricate relationship between cancerous and autoimmune processes,which remains incompletely understood. Several theories have been proposed to explain the pathogenesis of PNP correlated with Castleman disease (10). The first theory focuses on self-antibodies secreted by Castleman disease tumors, which are critical components of desmosomes that maintain cell-cell adhesion in the epidermis (11). These antibodies included desmosome proteins like desmoglein 1 and 3, as well as plakin family proteins. This antigen-antibody binding reaction leads to the formation of blisters in the mouth and skin, which are characteristics of PNP (3). Skin biopsies and immunoprecipitation tests are essential for diagnosing PNP, and help guide treatment decisions (3).

The activation of T-cells, particularly cytoxic T-cells, may have an important role in the patients with negative autoantibodies. CD8 + T-lymphocytes may contribute to the progression of bronchiolitis, and BO is the most worrying condition in patients with PNP associated with CD (3, 12). Pulmonary involvement could persist also after tumour resection and gradually progresses to respiratory failure and death. In this report, we also monitored relative immune indicators (Table 1), and found dynamic changes of litre of antinuclear antibody, IL-6 level and CD4/CD8 ratio. The immune findings may explain that, although the tumor lesions were surgically removed in a timely manner, the childs oral ulcers alleviated, but the skin conditions worsened.

Virus infection may also contribute to the worsening of mucosal involvement. In MCD patients, about half are prone to human herpesvirus 8 (HHV-8) infection, which mainly produces a functional analog of IL-6 (12). IL-6 promotes B-cell proliferation and survival, increasing autoantibody production and subsequent autoimmune manifestations, contributing to the systemic inflammatory response and immune dysregulation in PNP (13). Unusually, our patient was found to be infected with human herpesvirus 7 (HHV-7), which remains an area requiring further investigation.

In the present case, the dramatic worsening of mucocutaneous symptoms following an acute allergic reaction drew our attention. According to the literature, certain allergens may provoke or exacerbate pemphigus symptoms due to the immunogenic properties, including medications or environmental factors (14). The interplay between these allergic triggers and the autoimmune processes in PNP remains complex. On the other hand, patients with pemphigus may also exhibit a higher incidence of allergic reactions compared to the general population (15). The underlying mechanisms could be multifactorial, involving both the disease itself and the treatments commonly employed. Allergic reactions can manifest in various forms, complicating the clinical picture and potentially exacerbating the pemphigus condition (16).

Additionally, the pathology of PNP associated with CD is also corelated with the tumor itself. Castleman disease can be divided into hyaline vascular or plasmacytic and mixed types according to distinctive lymphoid architectural changes in nodal compartments. Various clinical presentations may be correlated with different underlying pathophysiological mechanisms. For instance, TAFRO syndrome, characterized by thrombocytopenia, anasarca, fever, renal dysfunction, and organomegaly, is a subtype of idiopathic multicentric Castleman disease and offers a specific theoretical framework. Scarcely, UCD and POEMS (Polyneuropathy, organomegaly, endocrinopathy, monoclonal protein, and skin changes)-associated MCD are caused by somatic mutations in monoclonal stromal and plasma cells (12, 17).

### 5.3 Therapeutic approach

The main treatment strategy focuses on treating the underlying Castleman disease to alleviate PNP symptoms. Surgical resection often cures unicentric CD. However, multicentric CD requires a multidisciplinary approach that includes systemic therapies, immunosuppressive agents, and surgical interventions (6). Long-term immunosuppressive therapy, which includes medications like rituximab, prednisolone, and azathioprine, is usually necessary to manage the autoimmune process associated with PNP. When used in combination with other systemic therapies, rituximab has been shown to lead to sustained remission in patients with PNP, highlighting its efficacy in managing mucosal lesions (18). Tocilizumab, an IL-6 receptor antagonist, can also effectively reduce systemic inflammation and control disease activity.

In cases where the Castleman disease tumor is resectable, surgery can benefit 90% of patients (9). UCD patients with PNP was recognized as an independent risk factor, often complicated by concurrent BO, with respiratory failure accounting for 77.8% of death (9). Additionally, intralesional rituximab, a local immunomodulatory treatment, has shown promise in treating recalcitrant mucosal lesions in PNP, providing a practical and economically feasible alternative to aggressive systemic therapies (1, 6). Furthermore, the presence of Castleman disease alongside other autoimmune conditions, such as systemic lupus erythematosus (SLE), highlights the necessity for tailored treatment strategies, which may include immunosuppressive agents, surgery, and supportive care (19). In this case, the child had low levels of CD4 T cells (Table 1) and significant side effects from steroids. Therefore, we faced the challenge of balancing immune strength while treating the underlying disease.

### 5.4 Prognosis and follow-up

PNP induced by Castleman disease, is a rare condition with a higher risk of mortality due to severe mucocutaneous or pulmonary complications. Univariate analysis indicated that both PNP and the elevation of C-reactive protein (CRP) were negative indicators of survival in patients with Castleman disease (12). Additionally, patients with PNP often succumb to severe infections resulting from their immunosuppressive treatments, making the management of PNP more challenging. Long-term follow-up and a multidisciplinary approach are essential for managing relapses and complications as well as monitoring disease activity and adjusting therapeutic strategies based on clinical response to optimize outcomes.

In this report, the girl received sequential follow-up and multidisciplinary collaborative treatment, overcoming the mucocuteneous symptoms gradually. However, after 3 months she developed dyspnea and the imaging findings suggested a trend towards BO, which arised great concern to us and requires further follow-up. In conclusion, we emphasize the importance of considering PNP in patients who present with persistent oral ulcers.

## Data Availability

The original contributions presented in the study are included in the article/Supplementary Material, further inquiries can be directed to the corresponding author.
